# Retraction Note: Oxycodone versus morphine for analgesia after laparoscopic endometriosis resection

**DOI:** 10.1186/s12871-021-01540-1

**Published:** 2021-12-11

**Authors:** Lijun Niu, Lihong Chen, Yanhua Luo, Wenkao Huang, Yunsheng Li

**Affiliations:** 1 Department of Anesthesiology, The First Affiliated Hospital, Sun Yat-sen University, No.58, Zhongshan 2nd Road, Guangzhou, 510080 China; 2 Department of Anesthesiology, The Six Affiliated Hospital, Sun Yat-sen University, No. 26, Erheng Road, Guangzhou, 510655 China; 3 Department of Anesthesiology, Zhongshan Ophthalmic Center, Sun Yat-Sen University, No.54 Xianlie South Road, Guangzhou, 510060 China; 4 Department of Anesthesiology, Nanfang Hospital, Southern Medical University, 1838 Guangzhou Avenue North, Guangzhou, China

**Retraction Note: BMC Anesthesiol 21, 194 (2021)**

**https://doi.org/10.1186/s12871-021-01417-3**

The Editor has retracted this article because after obtaining approval for this clinical trial from the Institutional Ethical Review Committee for Clinical Trials of The First Affiliated Hospital of Sun Yat-sen University the authors made changes to the protocol without obtaining the Committee's approval. All authors agree to this retraction.

